# Risk-related short-term clinical outcomes after transcatheter aortic valve implantation and their impact on early mortality: an analysis of claims-based data from Germany

**DOI:** 10.1007/s00392-022-02009-y

**Published:** 2022-03-24

**Authors:** Niklas Schofer, Elke Jeschke, Janine Kröger, Henning Baberg, Volkmar Falk, Jan F. Gummert, Christian W. Hamm, Martin Möckel, Alina Goßling, Jürgen Malzahn, Christian Günster, Stefan Blankenberg

**Affiliations:** 1 Department of General and Interventional Cardiology, University Heart Center Hamburg, Martinistrasse 52, 20246 Hamburg, Germany; 2 German Centre for Cardiovascular Research, DZHK, Partner Site Hamburg/Kiel/Lübeck, Hamburg, Germany; 3 Research Institute of the Local Health Care Funds, Berlin, Germany; 4 Department of Cardiology and Nephrology, Helios Klinikum, Berlin-Buch, Berlin, Germany; 5 Department of Cardiothoracic and Vascular Surgery, Deutsches Herzzentrum Berlin, Berlin, Germany; 6 Department of Cardiovascular Surgery, Charité Universitätsmedizin Berlin, Berlin, Germany; 7 German Centre for Cardiovascular Research, DZHK, Partner Site Berlin, Berlin, Germany; 8 Department of Health Science and Technology, ETH Zurich, Zürich, Switzerland; 9 Clinic for Thoracic and Cardiovascular Surgery, Heart and Diabetes Center NRW, Ruhr University Bochum, Bad Oeynhausen, Germany; 10 Medical Clinic I, University of Giessen and Campus Kerckhoff, Giessen/Bad Nauheim, Germany; 11 Division of Emergency Medicine and Chest Pain Units, Department of Cardiology, Campus Virchow-Klinikum and Mitte, Charité-Universitätsmedizin Berlin, Berlin, Germany; 12 Federal Association of the Local Health Care Funds (AOK), Baden-Württemberg, Germany

**Keywords:** Aortic stenosis, TAVI, Risk-related outcomes, Claims-based data

## Abstract

**Objectives:**

We aimed to define and assess risk-specific adverse outcomes after transcatheter aortic valve implantation (TAVI) in an all-comers patient population based on German administrative claims data.

**Methods:**

Administrative claims data of patients undergoing transvascular TAVI between 2017 and 2019 derived from the largest provider of statutory health-care insurance in Germany were used. Patients’ risk profile was assessed using the established Hospital Frailty Risk (HFR) score and 30-day adverse events were evaluated. Multivariable logistic regression models were applied to investigate the relation of patients’ risk factors to clinical outcomes and, subsequently, of clinical outcomes to mortality.

**Results:**

A total of 21,430 patients were included in the analysis. Of those, 51% were categorized as low-, 37% as intermediate-, and 12% as high-risk TAVI patients according to HFR score. Whereas low-risk TAVI patients showed low rates of periprocedural adverse events, TAVI patients at intermediate or high risk suffered from worse outcomes. An increase in HFR score was associated with an increased risk for all adverse outcome measures. The strongest association of patients’ risk profile and outcome was present for cerebrovascular events and acute renal failure after TAVI. Independent of patients’ risk, the latter showed the strongest relation with early mortality after TAVI.

**Conclusions:**

Differentiated outcomes after TAVI can be assessed using claims-based data and are highly dependent on patients’ risk profile. The present study might be of use to define risk-adjusted outcome margins for TAVI patients in Germany on the basis of health-insurance data.

**Graphical abstract:**

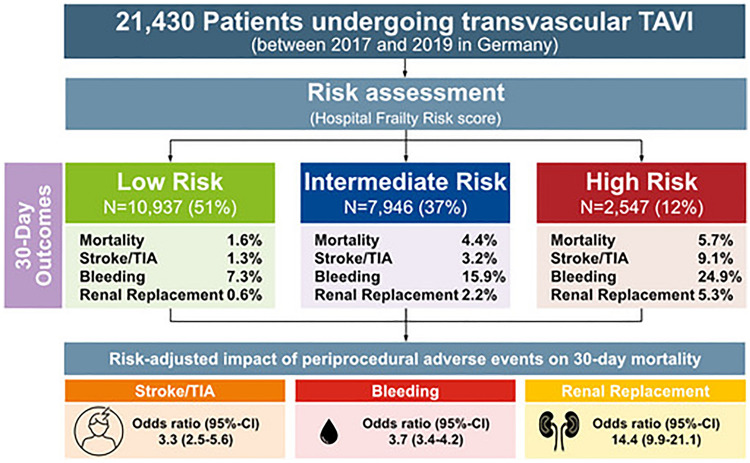

**Supplementary Information:**

The online version contains supplementary material available at 10.1007/s00392-022-02009-y.

## Introduction

Within the last decade there has been an enormous and continuous increase in the use of Transcatheter Aortic Valve Implantation (TAVI) for treatment of aortic stenosis (AS) in industrialized countries [1, 2]. In fact, in Germany as well as in the US TAVI has outnumbered surgical aortic valve replacement, and, thus, has become the first-choice therapy for the majority of patients suffering from severe AS. This development is accompanied by a broad fundament of randomized controlled trials (RCT) evaluating the safety and efficacy of TAVI as a treatment modality for AS patients across the whole risk spectrum [3–9]. Accordingly, use of TAVI has been expanded from treatment of inoperable AS patients in the early period towards treatment of AS patients at lower risk within more recent years. However, while RCT data is undoubtedly the gold standard for comparison of a new therapeutic concept against standard therapy, there is a significant difference in characteristics between those highly selected patients included in RCTs and those treated in daily practice. Hence, a substantial amount of TAVI patients is not adequately represented in current RCT data and, thus, the outcome of such TAVI patients is not sufficiently evaluated [10, 11]. To fill this gap in knowledge, claims-based health insurance data appears to be a valuable source, as it allows for outcome assessment among all TAVI patients. Such data could also be used to define generally accepted outcome margins for patients undergoing TAVI. Therefore, the aim of the present study was to investigate patients’ characteristics as well as to assess risk-specific, differentiated clinical outcomes after transvascular TAVI in a large patient cohort based on health-insurance claims in Germany.

## Methods

### Study design and study population

For this observational study, we used anonymized nationwide administrative claims data of the *Allgemeine Ortskrankenkasse* (AOK). The AOK provides health care insurance for approximately 30 percent of the German population and is the largest nation-wide provider of statutory health care insurance in Germany. We evaluated billing data for inpatient treatment, including diagnoses, procedures and length of stay, as well as patient data, including age, gender, insurance status (i.e., continued/terminated AOK membership) and survival. Diagnoses were encoded according to the International Classification of Diseases (ICD-10) [12]. Procedures were documented using the German version of the International Classification of Procedures in Medicine (ICPM), the OPS code [13]. Healthcare and health insurance providers jointly issue binding guidelines for coding of diagnoses and procedures in German hospitals. Hospital billing data are thoroughly checked by the Medical Review Board of the Health Insurance Funds and are returned to hospitals for correction if necessary.

We included patients who were aged 20 years or older, insured by the AOK and received endovascular TAVI (until 2017: OPS 5-35a.00; from 2018 on: OPS 5-35a.03 and 5-35a.04) between January 2017 and December 2019. Patients were excluded from the study if they had a primary diagnosis of “Endocarditis” (ICD-10 code: I33) or “Aortic (valve) insufficiency” (ICD-10 code: I35.1), or received “Other valve interventions” (OPS 5-35a, without 5-35a.0 and 5-35a.1).

### Risk stratification according to hospital frailty risk score

Patients’ risk profile was assessed by the Hospital Frailty Risk (HFR) score based on 109 ICD-10 3-digit-codes with predefined score points per diagnosis [14]. For each patient, we calculated the HFR score based on hospital diagnoses during the index case and within 3 months prior to index procedure. Due to the lack of information on the status “present on admission” in our database, some modifications of the HFR score were made. “Cerebral infarction” (ICD-10 code I63) and “Transient cerebral ischemic attacks and related syndromes” (G45) were only used for HFR score assessment, if the diagnosis was present in the index case without OPS code. “Delirium” (ICD-10 code F05), “Somnolence, stupor and coma” (R40), “Other septicemia” (A41) and “Acute renal failure” (N17) were only used for HFR score assessment if the diagnosis was present 3 months prior to index procedure. The prevalence of ICD-10 codes for the variables contributing two or more points to the creation of HFR score is shown in Supplementary Table 1. According to the literature the HFR score was categorized into 3 groups [low risk (< 5 points), intermediate risk (5–15 points) and high risk (> 15 points)] [15].

### Outcomes

The endpoints in the analysis were adverse events following index procedure, corresponding to the definitions of hospital quality indicators for TAVI, which were developed by the Research Institute of the Local Health Care Funds (WIdO) [16]. Outcomes were all-cause mortality within 30 days, myocardial infarction within 30 days, stroke or TIA within 30 days, bleeding within 7 days, access-related vascular complication within 7 days, permanent device implantation within 30 days, and acute renal failure with need for dialysis within 30 days. In addition, the six outcomes excluding all-cause mortality (also termed periprocedural adverse events) were also investigated as predictors of all-course mortality.

### Statistical analysis

Descriptive statistics including medians, inter-quartile ranges (IQRs), and proportions were used to describe the study sample. Baseline patient characteristics were compared using Pearson's chi-squared test for categorical variables and the Mann–Whitney *U* test for continuous measures. Subgroup analyses were performed for frailty risk categories, as well as for year of intervention.

C-statistics were used to calculate the predictive value of the HFR score. Multivariable logistic regression models were estimated, first, to evaluate the effect of HFR score points on outcomes, adjusting for patient age and gender, and second, to model the odds of 30-day mortality as a function of the separate periprocedural adverse events, adjusting for HFR score category. Third, multivariable logistic regression was used to determine factors associated with specific outcomes. Patient age, gender, body mass index (BMI), comorbidities, antithrombotic medication and treatment prior to surgery (i.e., myocardial infarction, stroke, percutaneous coronary intervention (PCI), heart surgery, dialysis, and aortic valve replacement) were included as independent patient-related variables. Comorbidities were defined using the Elixhauser conditions [17]. These include 31 acute and chronic diseases which we implemented using the coding algorithm by Quan et al. [18] (see Table 1). Some minor deviations from the Elixhauser conditions included separated BMI categories (< 30, 30–34, 35–39, ≥ 40 kg/m^2^) rather than the variable for obesity. Similarly, cardiac arrhythmia was split into atrial fibrillation and other cardiac arrhythmias. In addition, coronary heart disease, NYHA stage (IV vs. I–III), syncope, mitral insufficiency, tricuspid insufficiency, and pulmonary hypertension were included as independent variables. All comorbidities were entered as separate dichotomous variables. Patient age was entered as a continuous variable. Adjusted odds ratios (OR) and 95% confidence intervals (CI) were calculated. We used cluster-robust standard errors to account for clustering of patients in hospitals. Patient records were censored if AOK membership ended and none of the respective adverse events occurred during the follow-up period. All analyses were performed using STATA 16.0 (StataCorp LP, College Station, Texas).

**Table 1:** Baseline characteristics stratified by patients’ risk profile according to HFR score

Characteristic	All patients	low risk (HFR score < 5)	intermediate risk (HFR score 5–15)	high risk (HFR score > 15)	*p* value^1^
Patients, *N* (%)	21,430 (100)	10,937 (51.04)	7946 (37.08)	2547 (11.89)	
Age (y), median (IQR)	82 (78–85)	81 (78–84)	82 (79–86)	83 (79–86)	** < 0.001**
Female sex, n (%)	11,919 (55.62)	5783 (52.88)	4552 (57.29)	1584 (62,19)	** < 0.001**
Comorbidities (Elixhauser) ^2^, *n* (%)					
Congestive heart failure	16,388 (76.47)	7739 (70.76)	6406 (80.62)	2243 (88.06)	** < 0.001**
Atrial fibrillation	10,532 (49.15)	4523 (41.36)	4,326 (54.44)	1683 (66,08)	** < 0.001**
Other cardiac arrhythmia	3494 (16.30)	1866 (17.06)	1278 (16.08)	350 (13.74)	** < 0.001**
Peripheral vascular disorders	5282 (24.65)	2275 (20.80)	2126 (26.76)	881 (34.59)	** < 0.001**
Hypertension	19,310 (90.11)	9799 (89.59)	7142 (89.88)	2369 (93.01)	** < 0.001**
Paralysis	691 (3.22)	24 (0.22)	255 (3.21)	412 (16.18)	** < 0.001**
Neurological disorders	989 (4.62)	150 (1.37)	442 (5.56)	397 (15.59)	** < 0.001**
Chronic pulmonary disease	3.776 (17.62)	1612 (14.74)	1,550 (19.51)	614 (24.11)	** < 0.001**
Diabetes mellitus	7962 (37.15)	3641 (33.29)	3156 (39.72)	1165 (45.74)	** < 0.001**
Hypothyroidism	3713 (17.33)	1638 (14.98)	1464 (18.42)	611 (23.99)	** < 0.001**
Liver disease	710 (3.31)	261 (2.39)	293 (3.69)	156 (6.12)	** < 0.001**
Solid tumor without metastasis	524 (2.45)	218 (1.99)	213 (2.68)	93 (3.65)	** < 0.001**
Rheuma	758 (3.54)	312 (2.85)	315 (3.96)	131 (5.14)	** < 0.001**
Coagulopathy	1666 (7.77)	450 (4.11)	757 (9.53)	459 (18.02)	** < 0.001**
Obesity, BMI (kg/m^2^)	3389 (15.81)	1327 (14.14)	1319 (16.60)	523 (20.53)	** < 0.001**
30–34	2000 (9.33)	958 (8.76)	772 (9.72)	270 (10.60)	
35–39	870 (4.06)	369 (3.37)	343 (4.32)	158 (6.20)	
≥ 40	519 (2.42)	220 (2.01)	204 (2.57)	95 (3.73)	
Weight loss	683 (3.19)	94 (0.86)	285 (3.59)	304 (11.94)	** < 0.001**
Fluid and electrolyte disorders	7210 (33.64)	1687 (15.42)	3781 (47.58)	1742 (68.39)	** < 0.001**
Iron deficiency anemia	1464 (6.83)	370 (3.38)	687 (8.65)	407 (15.98)	** < 0.001**
Drug abuse	117 (0.55)	28 (0.26)	58 (0.73)	31 (1.22)	** < 0.001**
Depression	935 (4.36)	224 (2.05)	379 (4.77)	332 (13.03)	** < 0.001**
Renal failure	10,451 (48.77)	3812 (34.85)	4801 (60.42)	1838 (72.16)	** < 0.001**
Comorbidities (other), *n* (%)					
Coronary heart disease	7964 (37.16)	3855 (35.25)	3019 (37.99)	1090 (42.80)	** < 0.001**
Mitral regurgitation	4911 (22.92)	1909 (17.45)	2159 (27.17)	843 (33.10)	** < 0.001**
Ticuspid regurgitation	3089 (14.41)	1159 (10.60)	1387 (17.46)	543 (21.32)	** < 0.001**
Pulmonary hypertension	5531 (25.81)	2393 (21.88)	2304 (29.00)	834 (32.74)	** < 0.001**
Symptomes, *n* (%)					
NYHA IV	3837 (17.90)	921 (8.42)	1878 (23.63)	1038 (40.75)	** < 0.001**
Syncope	807 (3.77)	158 (1.44)	410 (5.16)	239 (9.38)	** < 0.001**
Treatment prior to index surgery, *n* (%)					
Antithrombotic medication	12,390 (57.82)	6069 (55.49)	4740 (59.65)	1581 (62.07)	** < 0.001**
Myocardial infarction less than 1 y before TAVI	1266 (5.91)	480 (4.39)	552 (6.95)	234 (9.19)	** < 0.001**
Stroke less than 1 y before TAVI	652 (3.04)	202 (1.85)	269 (3.39)	181 (7.11)	** < 0.001**
PCI less than 90 d before TAVI	1656 (7.73)	637 (5.82)	713 (8.97)	306 (12.01)	** < 0.001**
Heart surgery less than 1 y before TAVI	780 (3.64)	351 (3.21)	316 (3.98)	113 (4.44)	**0.002**
Dialysis less than 1 y before TAVI	667 (3.11)	191 (1.75)	324 (4.08)	152 (5.97)	** < 0.001**
Aortic valve replacement less than 10 y before TAVI	368 (1.72)	208 (1.90)	126 (1.59)	34 (1.33)	0.073
Prozedural characteristics, *n* (%)					
TAVI devices^3^					0.524
Balloon	6112 (41.36)	3163 (41.49)	2243 (41.48)	706 (40.11)	
Self-expandable	8665 (58.64)	4460 (58.51)	3151 (58.42)	1054 (59.89)	
Use of a cerebral protection device	1294 (6.04)	723 (6.61)	438 (5.51)	133 (5.22)	**0.001**

Significant values are in bold

Significance defined as *p* < 0.0013 after Bonferroni correction

*BMI* body mass index, *HFR* hospital frailty risk, *NYHA* New York Heart Association classification, *PCI* percutaneous coronary intervention, *TAVI* transcatheter Aortic Valve Implantation

^1^Pearson’s Chi^2^ test or Median test for age

^2^Other comorbidities included in the analysis but with a frequency < 2.0% in the whole cohort are not shown (pulmonary circulation disorders, blood loss anemia, peptic ulcer disease excluding bleeding, alcohol abuse, drug abuse, psychoses, AIDS/HIV, solid tumor, lymphoma)

^3^Data only available for patients treated in years 2018 and 2019 (*N* = 14,777)

## Results

### Risk score distribution and patients’ characteristics

**Fig. 1:**
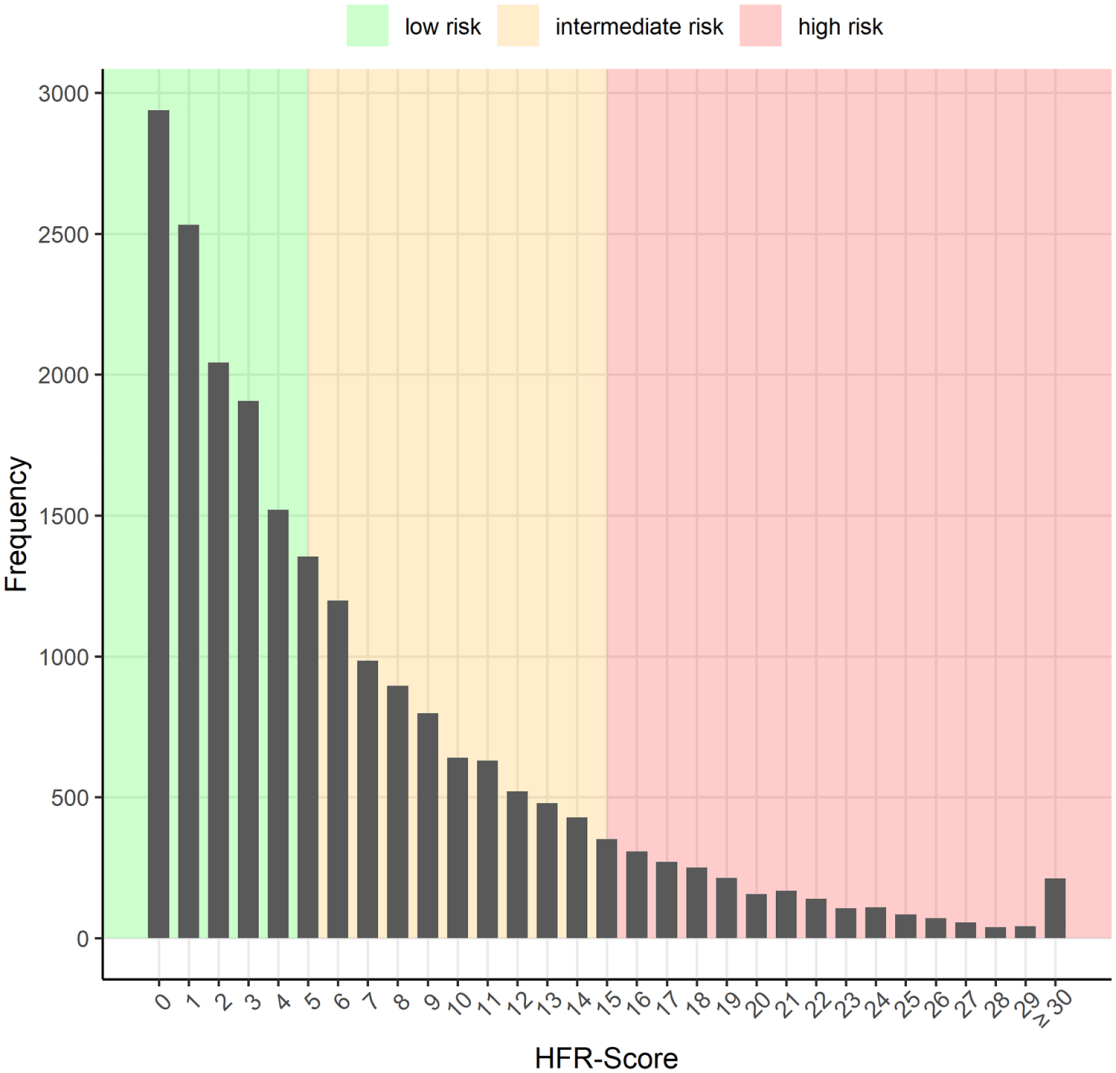
Distribution of HFR score points across the study population

Figure 1 illustrates the distribution of HFR score points across the study population. A total of 21,430 patients were included in the analyses. Of those, 51% (*N* = 10,937) were assigned to the low risk category, 37% (*N* = 7946) to the intermediate risk and 12% (*N* = 2547) to the high risk category, respectively. The median HFR score among the study population was 4.8 points (IQR 1.8–9.8) and significantly decreased during the observation period (median HFR score according to year of TAVI [2017 vs. 2018 vs. 2019: 5.0 vs. 4.9 vs. 4.6, *p* = 0.003; Supplementary Fig. 1).

Table 1 shows baseline as well as procedural characteristics of the study population stratified by risk category. Compared to patients at lower risk, high-risk patients were older and more often female. Moreover, as expected, high-risk patients presented with a higher burden of relevant cardiovascular and non-cardiovascular comorbidities. There was no difference in use of balloon- vs. self-expandable TAVI devices between the risk categories. The rate of use of cerebral protection devices during TAVI was low among the study population, but was more frequent in patients at lower risk.

### Risk-specific 30-day outcomes

Risk-specific clinical outcomes at 30 days after TAVI are given in Table 2. Patients in the low-risk category had the lowest 30-day mortality rate with 1.6% vs. 4.4% and 5.7% for those in the intermediate- and high-risk category, respectively (*p* < 0.001). Likewise, 30-day rates of stroke, myocardial infarction, acute renal failure with need for dialysis and permanent device implantation as well as bleeding and vascular complications within 7 days following the procedure were all in favor for TAVI patients in the low-risk category.

**Table 2:** Outcomes within 30 days after TAVI stratified by patients’ risk profile according to HFR score

Endpoint	All patients, *n* = 21,430	Low risk (HFR score < 5), *n* = 10,973	Intermediate risk (HFR score 5–15), *n* = 7,946	High risk (HFR score > 15), *n* = 2,547	*p* value
Mortality, *n* (%)	665 (3.1)	175 (1.6)	346 (4.4)	144 (5.7)	** < 0.001**
Myocardial infarction, *n* (%)	82 (0.4)	36 (0.3)	33 (0.4)	13 (0.5)	0.111
Stroke or TIA, *n* (%)	625 (2.9)	137 (1.3)	255 (3.2)	233 (9.1)	** < 0.001**
Bleeding^1^, *n* (%)	2.696 (12.6)	803 (7.3)	1.260 (15.9)	633 (24.9)	** < 0.001**
Access-related vascular complication^1^, *n* (%)	457 (2.1)	201 (1.8)	182 (2.3)	74 (2.9)	** < 0.001**
Permanent device implantation, *n* (%)	3,008 (14.0)	1,386 (12.7)	1,184 (14.9)	438 (17.2)	** < 0.001**
Acute renal failure with need for dialysis, *n* (%)	375 (1.7)	63 (0.6)	178 (2.2)	134 (5.3)	** < 0.001**

Significant values are in bold

Censoring taken into account

*HFR* hospital frailty risk, *TAVI* Transcatheter Aortic Valve Implantation

^1^Within 7 days

Table 3 displays the association of patient’s risk-profile according to HFR score and risk for adverse outcome. An increase in HFR score points was related to an increase in risk for all adverse outcome measures. The strongest association of patients’ risk and outcome was present for periprocedural stroke [OR per increase in HFR score point: 1.11 (95% CI 1.10–1.12)], followed by acute renal failure [OR per increase in HFR score point: 1.11 (95% CI 1.09–1.12)] and bleeding [OR per increase in HFR Score point: 1.08 (95% CI 1.07–1.09)]. No difference was found between risk-specific outcomes according to year of treatment (supplementary Table 2). The c-statistic for mortality within 30 days after TAVI using only the HFR score was 0.67. The discriminatory value of the HFR score to predict each single adverse event is given in supplementary Table 3.

**Table 3:** Impact of patients’ risk profile according to HFR score on outcomes within 30 days after TAVI

Outcome	Impact of HFR score (per 1-point increase)
	Adjusted OR (95%-CI)
Mortality	1.06 (1.05–1.07)
Myocardial infarction	1.04 (1.02–1.06)
Stroke or TIA	1.11 (1.10–1.12)
Bleeding^1^	1.08 (1.07–1.09)
Access-related vascular complication^1^	1.04 (1.02–1.05)
Permanent device implantation	1.03 (1.02–1.04)
Acute renal failure with need for dialysis	1.11 (1.09–1.12)

Models were adjusted for patient age and gender

*TIA* transient ischemic attack, *CI* confidence interval; all other abbreviations as in Table 1

^1^Within 7 days, all other adverse events within 30 days

### Risk factors for adverse outcomes 30 days after TAVI

Table 4 shows predictors for 30-day mortality according to multivariable analyses for the overall cohort as well as for each patient risk category separately. Coagulopathy was found to provide the strongest association with 30-day mortality, followed by preexisting liver disease, NYHA functional class IV, prior dialysis, paralysis, fluid and electrolyte disorders, peripheral vascular disease, and age. In contrast, BMI 30–34 kg/m^2^ was independently associated with survival at 30 days after TAVI.

**Table 4:** Risk factors for mortality within 30 days after TAVI

Risk factor*	Adjusted OR (95%-CI)			
	All patients^1^	Low risk (HFR score < 5)	Intermediate risk (HFR score 5–15)	High risk (HFR score > 15)
Age (y)	1.04 (1.02–1.06)	1.05 (1.02–1.08)	1.03 (1.01–1.06)	1.06 (1.01–1.10)
Comorbidities (Elixhauser)				
BMI 30–34 kg/m^2^	0.57 (0.42–0.76)	–	–	0.31 (0.17–0.57)
Peripheral vascular disorders	1.25 (1.03–1.50)	1.61 (1.12–2.31)	–	–
Liver disease	2.36 (1.74–3.19)	2.28 (1.28–4.06)	2.64 (1.69–4.11)	2.14 (1.24–3.70)
Paralysis	1.78 (1.20–2.65)	–	3.22 (1.99–5.22)	–
Coagulopathy	5.10 (4.14–6.30)	14.86 (10.49–21.05)	5.20 (3.87–7.00)	1.80 (1.29–2.51)
Fluid and electrolyte disorders	1.49 (1.23–1.82)	1.59 (1.07–2.39)	1.46 (1.14–1.85)	–
NYHA IV	2.30 (2.41–3.71)	4.94 (3.29–7.43)	3.10 (2.46–3.90)	1.75 (1.26–2.44)
Dialysis less than 1 y before the surgery	1.99 (1.36–2.90)	2.98 (1.29–6.97)	2.17 (1.41–3.36)	–

Abbreviations as in Table 1

^*^Models were adjusted for patient age, gender, BMI (< 30 vs. 30–34, 35–39, ≥ 40 kg/m^2^), all 31 Elixhauser comorbidities, antithrombotic medication, interventions prior to surgery (i.e., myocardial infarction, stroke, percutaneous coronary intervention, heart surgery, dialysis, and aortic valve replacement), coronary heart disease, NYHA stage (IV vs. I–III), syncope, mitral insufficiency, tricuspid insufficiency, and pulmonary hypertension

Only significant risk factors for are shown

Whereas coagulopathy showed the strongest association with early mortality after TAVI in low- and intermediate-risk patients, among high-risk TAVI patients preexisting liver disease was the strongest risk factor for 30-day mortality. Moreover, BMI 30–34 kg/m^2^ was strongly associated with short-term survival in these patients, but no such association was present among the low- and intermediate-risk TAVI patient subgroup.

Risk-specific, independent predictors of periprocedural stroke or TIA, acute renal failure, bleeding or vascular complications are presented in supplementary Tables 4–6.

### Risk-adjusted association of periprocedural adverse events and early mortality after TAVI

**Fig. 2:**
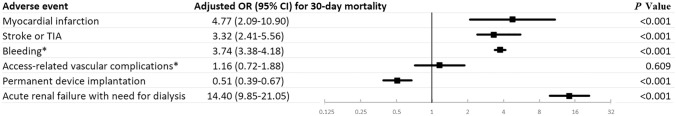
Risk-adjusted impact of periprocedural adverse events on 30-day mortality. *Within 7 days, all other adverse events within 30 days. Multivariable logistic regression model was used to calculate ORs and 95% CIs. Model is adjusted for HFR risk categories

In 91% (*n* = 606) of patients who died within 30 days after TAVI (*N* = 665), at least one adverse periprocedural clinical event occurred. The risk-adjusted, independent impact of single periprocedural events on 30-day mortality is illustrated in Fig. 2. The highest adjusted risk for 30-day mortality was present for patients with acute renal failure who were in need of dialysis after TAVI followed by periprocedural myocardial infarction and stroke or TIA. No independent impact on 30-day mortality was seen for vascular complications. In contrast, after adjustment for patients’ risk as well as all other periprocedural events, permanent pacemaker implantation within 30 days after TAVI was protective with regard to 30-day mortality.

## Discussion

In the present study we assessed characteristics as well as differentiated, risk-specific short-term outcomes in a large AS patient cohort undergoing TAVI based on data derived from current public health-insurance claims in Germany. The principal findings of the study are as follows: (1) approximately half of the TAVI patients using the statutory public health-insurance system in the study period can be considered low-risk patients according to HFR score. The other half of TAVI patients present at increased (intermediate or high) risk. (2) While low-risk TAVI patients show low rates of periprocedural adverse events and mortality at 30 days after the procedure, TAVI patients at intermediate or high risk suffer from a substantially higher rate of periprocedural adverse events and short-term mortality. (3) The strongest association of patients’ risk profile and outcome is present for cerebrovascular events and acute renal failure with need for dialysis after TAVI. (4) Independent of patients’ risk, acute renal failure with need for dialysis showed the strongest relation with early mortality after TAVI by far.

Since TAVI has been adopted as a treatment modality for patients with AS, the number of procedures in Germany has increased from 2500 in the year 2009 to almost 25,000 in the year 2019 [2, 19, 20]. During this period, characteristics of TAVI patients have changed fundamentally from only few selected high-risk patients with AS towards the vast majority of AS patients, including those at lower risk. In accordance with this development, in-hospital mortality after TAVI has decreased continuously over time [19, 20]. The present study provides a comprehensive overview of current characteristics and outcomes in more than one third (35%) of TAVI patients in Germany. For the purpose of the study the patient cohort was categorized according to the HFR score in low-, intermediate-, or high-risk patients. Accuracy of this score for TAVI patients’ risk assessment based on claims-based data has been validated before [15]. By applying the HFR score, we could show that among a large cohort of patients utilizing the statutory public-health system in Germany about half of the individuals currently undergoing TAVI can be considered low-risk patients, whereas the other half remains at increased risk. Of note, median age of low-risk patients in the present study was 81 years, clearly indicating that low-risk by HFR score is not equivalent to low surgical risk, which can be estimated using established surgical risk score calculators, i.e., STS PROM or EuroSCORE II score. Although patients’ risk decreased during the observation period in the present study, in direct comparison to US claims-based data derived from 28,531 TAVI patients treated in the calendar year 2016, which was published by Kundi et al., patients’ risk was slightly higher in our study (present study vs. Kundi et al.: 51.0% vs. 52.4% low-risk patients, 11.9% vs. 8.1% high-risk patients; mean HFR score points 6.9 ± 6.7 vs. 6.3 ± 5.7) [15]. This is an interesting finding, as it demonstrates that even using large data sets of all-comer patients, there still may be a significant difference in patients’ characteristics between data derived from different health-care systems. Universally applicable score systems, like the ICD-10-based HFR score, might be helpful tools to adjust for these differences.

The present study is the first to provide information on differentiated outcomes in patients undergoing TAVI derived from health-care claims in Germany. Therefore, outcome parameters were defined using codes for certain diagnoses (ICD) and/or procedures (OPS). In general, this data source appears to be a valuable alternative to national registry data, as the latter is predominantly based on non-quality controlled, self-reported data. This applies especially to outcomes based on procedure codes, e.g., pacemaker implantation, which provide a high accuracy, very similar to trial-adjudicated data [21]. In contrast, when data of health-care providers is used, outcomes that are mainly based on diagnosis codes, e.g., bleeding, are known to have only limited validity compared to trial-adjudicated outcomes [21]. Accordingly, when comparing the outcome data demonstrated in the present study with TAVI outcomes assessed by the German Aortic Valve Registry (GARY), a large registry collecting self-reported data from centers performing TAVI in Germany, some differences become apparent. This can be seen with respect to the rate of permanent pacemaker implantation during index hospitalization after transvascular TAVI, which has been described with 9.6% for the year 2017 in the GARY registry compared to 14.2% for the same year according to our data set (data not shown in results) [20]. In contrast, in-hospital rate of vascular complications are more frequent according to the GARY data set (6.0% in 2017) in comparison with our data (2.4% in 2017, data not shown in results) and, hence, might be underestimated by solely using claims-based data [20]. However, regarding further important in-hospital outcomes, such as periprocedural stroke (year 2017 GARY vs. present study: 1.9% vs. 2.6, data not shown in results), myocardial infarction (year 2017 GARY vs. present study 0.2% vs. 0.4%, data not shown in results), and, most important, in-hospital mortality after transvascular TAVI (2017–2019 GARY vs. present study: 2.9% vs. 2.8%, data not shown in results) fewer discrepancies are present [19, 20].

The other important finding of the current study is the association of patients’ risk profile and periprocedural adverse events after TAVI. This not only accounts for early mortality, but, as demonstrated by our data, affects any periprocedural adverse outcome. Accordingly, the present study complements current evidence by demonstrating excellent procedural outcomes among an all-comer low-risk TAVI population [1, 19, 20]. Yet, TAVI patients at increased risk still suffer from worse outcome. The strongest relation of risk and outcome was present for cerebrovascular events and acute renal failure after TAVI. Moreover, the latter adverse event provided the by far strongest association with early mortality after TAVI. This data underlines two major points: First, it will be of utmost importance to thoroughly adjust for patient-related risk factors when outcome margins for TAVI patients’ are being derived based on health-insurance data. For this purpose, a risk-assessment tool should be utilized which has been validated in claims-based data sets, just like the HFR score used in this study. Second, with respect to short-term mortality, certain adverse periprocedural events after TAVI, e.g., renal failure, myocardial infarction, bleeding or cerebrovascular events, are “more severe” than others, e.g., permanent pacemaker implantation. Thus, avoiding the most severe adverse events after TAVI might have the strongest positive impact on survival early after TAVI.

Some limitations regarding the present study have to be acknowledged. First, as the present analysis is based on German health insurance data, our findings cannot be translated into health care systems of other countries. Second, although the present study is based on nationwide data of the largest healthcare insurance provider in Germany, there may be variations in terms of age, gender, social status, and morbidity between patients insured by different providers [22]. Third, the definition of clinical outcomes were based on ICD and OPS codes, which cannot be equated with outcomes assessed on the basis of clinical trials. Fourth, data on procedural characteristics is limited in the present study, thus we cannot differentiate between patients with transvascular, non-transfemoral (e.g., transaxillary, transcarotid or transcaval) TAVI and patients with transvascular, transfemoral TAVI, which is the standard access site for TAVI. Still, the proportion of transvascular, non-transfemoral TAVI can be assumed to be less than 5% according to recent data [1].

## Conclusions

The present study provides insights on short-term outcomes among all-comer patients currently undergoing TAVI in Germany solely based on data derived from health-insurance claims. We could demonstrate that clinical outcomes after TAVI are highly dependent on patients’ risk profile, with excellent results after TAVI in patients at low risk, but an increased risk for various adverse events after TAVI in patients at intermediate or high risk. Our approach could be used to derive risk-adjusted outcome margins for TAVI patients in Germany on the basis of health-insurance data.

## Supplementary Information

Below is the link to the electronic supplementary material.

Supplementary file1 (DOCX 32 kb)
