# Supplementary material for: Risk-related short-term clinical outcomes after transcatheter aortic valve implantation and their impact on early mortality: an analysis of claims-based data from Germany
Source: Clin Res Cardiol. 2022 Mar 24;111(8):934–43. doi: 10.1007/s00392-022-02009-y (PMC9334430; doi:10.1007/s00392-022-02009-y)
Supplement: Supplementary file 1 — Supplementary file1 (DOCX 32 kb) [file 392_2022_2009_MOESM1_ESM.docx]

**Supplement**

**Supplemantary Figure Legends**

**Supplementary Fig. 1**

Hospital Frailty Risk according to year of TAVI.

**Supplementary Table 1: List of ICD-10-codes, their prevalence, and the number of points that each variable contributes to the creation of the HFR score.**

| ICD-10 code | Description | Patient, n = 21,430, n (%) | Points |
| --- | --- | --- | --- |
| G81 | Hemiplegia | 540 (2.52) | 4.4 |
| G30 | Alzheimer’s disease | 120 (0.56) | 4.0 |
| I69 | Sequelae of cerebrovascular disease | 582 (2.72) | 3.7 |
| R29 | Other symptoms and signs involving the nervous and musculoskeletal systems (R29.6 Tendency to fall) | 784 (3.66) | 3.6 |
| N39 | Other disorders of urinary system (includes urinary tract infection and urinary incontinence) | 3,092 (14.43) | 3.2 |
| F05 | Delirium, not induced by alcohol and other psychoactive substances | 0 | 3.2 |
| W19 | Unspecified fall | 0 | 3.2 |
| S00 | Superficial injury of head | 163 (0.76) | 3.2 |
| R31 | Unspecified haematuria | 598 (2.79) | 3.0 |
| B96 | Other bacterial agents as the cause of diseases classified to other chapters  (secondary code) | 2,182 (10.18) | 2.9 |
| R41 | Other symptoms and signs involving cognitive functions and awareness | 340 (1,59) | 2.7 |
| R26 | Abnormalities of gait and mobility | 1,893 (8.83) | 2.6 |
| I67 | Other cerebrovascular diseases | 841 (3.92) | 2.6 |
| R56 | Convulsions, not elsewhere classified | 47 (0.22) | 2.6 |
| R40 | Somnolence, stupor, and coma | 0 | 2.5 |
| T83 | Complications of genitourinary prosthetic devices, implants, and grafts | 173 (0.83) | 2.4 |
| S06 | Intracranial injury | 69 (0.32) | 2.4 |
| S42 | Fracture of shoulder and upper arm | 35 (0.16) | 2.3 |
| E87 | Other disorders of fluid, electrolyte, and acid-base balance | 6,939 (32.38) | 2.3 |
| M25 | Other joint disorders, not elsewhere classified | 182 (0.85) | 2.3 |
| E86 | Volume depletion | 807 (3.77) | 2.3 |
| R54 | Senility | 182 (0.85) | 2.2 |
| F03 | Unspecified dementia | 558 (2.60) | 2.1 |
| W18 | Other fall on same level | 0 | 2.1 |
| Z75 | Problems related to medical facilities and other health care | 43 (0.20) | 2.0 |
| F01 | Vascular dementia | 217 (1.01) | 2.0 |
| S80 | Superficial injury of lower leg | 85 (0.40) | 2.0 |
| L03 | Cellulitis | 46 (0.21) | 2.0 |

Table presents the top 28 codes, each of which contributes >= 2 points

**Supplementary Table 2. Relationship between HFR category and year of TAVI for outcomes within 30 days.**

| **Endpoint** | **P value^1^** |
| --- | --- |
| Mortality | 0.162 |
| Myocardial infarction | 0.779 |
| Stroke or TIA | 0.390 |
| Bleeding^2^ | 0.126 |
| Access-related vascular complication^2^ | 0.890 |
| Permanent device implantation | 0.882 |
| Acute renal failure with need for dialysis | 0.443 |

Abbreviation: HFR, hospital frailty risk; TAVI, Transcatheter Aortic Valve Implantation

^1^Chi^2^ test; ^2^within 7 days

**Supplementary Table 3. Predictive value of the HFR score**

| **30-day Outcome** | **C-statistic** |
| --- | --- |
| Mortality | 0,67 |
| Myocardial infarction | 0,57 |
| Stroke or TIA | 0,74 |
| Bleeding^1^ | 0,68 |
| Access-related vascular complication | 0,57 |
| Permanent device implantation | 0,55 |
| Acute renal failure with need for dialysis | 0,77 |

Abbreviation as in Table 3.

^1^within 7 days, all other adverse events within 30 days

**Supplementary Table 4. Risk factors for stroke or TIA within 30 days after TAVI.**

|  | **Adjusted OR (95%-CI)** | | | |
| --- | --- | --- | --- | --- |
| **Risk factor*** | **All patients^1^** | **Low risk (< 5 HFR points)** | **Intermediate risk (5-15 HFR points)** | **High risk (> 15 HFR points)** |
| Age (y) | 1.04 (1.02-1.05) | 1.02 (1.003-1.04) | 1.03 (1.01-1.06) | - |
| **Comorbidities** |  |  |  |  |
| Atrial fibrillation | 1.55 (1.31-1.84) | - | - | - |
| Peripheral vascular disorders | 1.38 (1.11-1.70) | 1.41 (1.01-1.97) | - | - |
| Neurological disorders | 11.03 (8.85-13.75) | 7.65 (3.89-15.05) | 7.60 (5.20-11.11) | 7.67 (5.80-10.15) |
| Coagulopathy | 1.59 (1.25-2.03) | - | 1.63 (1.07-2.46) | - |
| Weight loss | 1.45 (1.05-2.00) | - | - | - |
| Fluid and electrolyte disorders | 1.55 (1.31-1.84) | - | - | 1.60 (1.20-2.11) |
| Depression | 1.81 (1.34-2.43) | 2.51 (1.17-5.34) | - | - |
| Dialysis less than 1 y before the surgery | 1.56 (1.09-2.25) | - | - | - |

Abbreviations as in Table 1.

*Models were adjusted for patient age, gender, BMI (<30 vs. 30-34, 35-39, ≥40 kg/m²), all 31 Elixhauser comorbidities, antithrombotic medication, interventions prior to surgery (i.e. myocardial infarction, stroke, percutaneous coronary intervention, heart surgery, dialysis, and aortic valve replacement), coronary heart disease, NYHA stage (IV vs. I-III), syncope, mitral insufficiency, tricuspid insufficiency, and pulmonary hypertension.

Only significant risk factors are shown.

**Supplementary Table 5. Risk factors for acute renal failure with need for dialysis within 30 days after TAVI.**

|  | **Adjusted OR (95%-CI)** | | | |
| --- | --- | --- | --- | --- |
| **Risk factor** | **All patients^1^** | **Low risk (< 5 HFR points)** | **Intermediate risk (5-15 HFR points)** | **High risk (> 15 HFR points)** |
| Atrial fibrillation | 1.59 (1.14-2.22) | - | 1.68 (1.02-2.77) | - |
| Other cardiac arrhythmia | 1.68 (1.12-2.52) | - | 2.14 (1.24-2.70) | - |
| BMI 30-34 kg/m² | 1.97 (1.16-3.33) |  | 2.27 (1.18-4.34) |  |
| Paralysis | 2.42 (1.57-3.74) | - | 3.86 (2.00-7.46) | - |
| Diabetes mellitus | 1.40 (1.16-1.69) | - | 1.37 (1.001-1.87) | - |
| Liver disease | 2.73 (1.72-4.32) | - | 3.12 (1.59-6.18) | 2.25 (1.20-4.20) |
| Coagulopathy | 10.84 (8.34-14.10) | 10.98 (10.74-35.80) | 10.81 (7.31-15.97) | 6.85 (4.75-9.88) |
| Fluid and electrolyte disorders | 3.06 (2.26-4.12) | 2.24 (1.13-4.43) | 2.65 (1.85-3.80) | 2.79 (1.74-4.47) |
| Coronary heart disease | 1.35 (1.09-1.69) | - | 1.56 (1.16-2.08) | - |
| NYHA IV | 2.37 (1.85-3.05) | 4.39 (2.58-7.47) | 2.07 (1.43-2.99) | 1.73 (1.24-2.40) |
| Renal failure | 1.77 (1.32-2.38) | - | - | 2.10 (1.31-3.36) |

Abbreviations as in Table 1.

*Models were adjusted for patient age, gender, BMI (<30 vs. 30-34, 35-39, ≥40 kg/m²), all 31 Elixhauser comorbidities, antithrombotic medication, interventions prior to surgery (i.e. myocardial infarction, stroke, percutaneous coronary intervention, heart surgery, dialysis, and aortic valve replacement), coronary heart disease, NYHA stage (IV vs. I-III), syncope, mitral insufficiency, tricuspid insufficiency, and pulmonary hypertension.

Only significant risk factors are shown.

**Supplementary Table 6. Risk factors for bleeding or vascular complications within 7 days after TAVI.**

|  | **Adjusted OR (95%-CI)** | | | |
| --- | --- | --- | --- | --- |
| **Risk factor** | **All patients^1^** | **Low risk (< 5 HFR points)** | **Intermediate risk (5-15 HFR points)** | **High risk (> 15 HFR points)** |
| Age (y) | 1.01 (1.003-1.02) | - | - | - |
| Female sex | 1.89 (1.71-2.08) | 1.83 (1.58-2.11) | 1.88 (1.60-2.21) | 1.83 (1.48-2.25) |
| **Comorbidities** |  |  |  |  |
| Peripheral vascular disorders | 1.46 (1.29-1.65) | 1.61 (1.36-1.91) | 1.35 (1.15-1.59) | 1.29 (1.04-1.61) |
| Paralysis | 1.60 (1.29-1.98) | - | 1.51 (1.13-2.03) | - |
| Neurological disorders | 1.24 (1.06-1.46) | - | - | - |
| Liver disease | 1.45 (1.18-1.78) | - | 1.41 (1.06-1.88) | 1.54 (1.02-2.33) |
| Solid tumor without metastasis | 1.38 (1.07-1.77) | - | 1.64 (1.15-2.33) | - |
| Rheuma | 1.27 (1.05-1.54) | - | - | 1.74 (1.21-2.49) |
| Coagulopathy | 4.72 (4.10-5.44) | 6.58 (4.99-8.68) | 4.18 (3.45-5.07) | 3.33 (2.65-4.19) |
| Weight loss | 1.97 (1.48-2.63) | 1.98 (1.05-3.73) | 1.70 (1.31-2.22) | 1.79 (1.15-2.77) |
| Fluid and electrolyte disorders | 1.58 (1.40-1.77) | 1.36 (1.11-1.66) | 1.32 (1.15-1.52) | 1.36 (1.07-1.74) |
| Drug abuse | 3.29 (2.27-4.76) | - | 2.52 (1.24-5.10) | 3.57 (1.92-6.61) |
| Coronary heart disease | 1.19 (1.09-1.29) | - | 1.26 (1.10-1.46) | 1.36 (1.06-1.78) |
| NYHA IV | 1.32 (1.18-1.48) | - | 1.27 (1.10-1.48) | - |
| Mitral valve insufficiency | 1.16 (1.01-1.33) | - | - | - |
| Syncope | 1.28 (1.05-1.56) | - | - | - |
| Myocardial infarction less than 1 y before the surgery | 1.41 (1.20-1.67) | 1.67 (1.24-2.26) | - | - |
| PCI less than 90 d before the surgery | 1.61 (1.34-1.92) | 2.04 (1.62-2.57) | 1.40 (1.14-1.73) | 1.44 (1.02-2.04) |
| Dialysis less than 1 y before the surgery | 1.94 (1.53-2.45) | 2.22 (1.47-3.36) | - | 2.25 (1.66-3.05) |

Abbreviations as in Table 1.

*Models were adjusted for patient age, gender, BMI (<30 vs. 30-34, 35-39, ≥40 kg/m²), all 31 Elixhauser comorbidities, antithrombotic medication, interventions prior to surgery (i.e. myocardial infarction, stroke, percutaneous coronary intervention, heart surgery, dialysis, and aortic valve replacement), coronary heart disease, NYHA stage (IV vs. I-III), syncope, mitral insufficiency, tricuspid insufficiency, and pulmonary hypertension.

Only significant risk factors are shown.
